# Supplementary material for: Differential roles of lysosomal cholesterol transporters in the development of C. elegans NMJs
Source: Life Sci Alliance. 2024 Jul 31;7(10):e202402584. doi: 10.26508/lsa.202402584 (PMC11291935; doi:10.26508/lsa.202402584)
Supplement: Supplementary file 2 [file LSA-2024-02584_TableS1.docx]

**Table S1. Strains, transgenes and constructs.**



| **Strain** | **Genotype** | **Transgene** | **Figure** | **Plasmid** | **Transgene construction notes** |
| --- | --- | --- | --- | --- | --- |
| KP3292 | *Punc-129-SNB-1::GFP, ttx::RFP(nuIs152)* | *Punc-129:SNB-1::GFP, ttx::RFP* | Figure 1I-1J |  |  |
| CZ333 | *Punc-25-SNB::GFP (juIs1) IV* | *Punc-25-SNB::GFP* |  |  |  |
| FCZ97 | *Punc-25-SNB::GFP (juIs1) IV；ncr-1(nr2022) X* | *Punc-25-SNB::GFP* | Figure 1B-1D, 1G-1H, 1K-1M, 2B-2D, 2F-2H and S3 |  |  |
| FCZ98 | *Punc-25-SNB::GFP (juIs1) IV; ncr-2(nr2023) III* |  |  |  |  |
| FCZ99 | *Punc-25-SNB::GFP (juIs1) IV ; ncr-2(nr2023) III ; ncr-1(nr2022) X* |  |  |  |  |
| FCZ426 | *heh-1(ok603) III; Punc-25-SNB::GFP (juIs1) IV* | *Punc-25-SNB::GFP* | Figure 4D |  |  |
| FCZ427 | *Punc-25-SNB::GFP (juIs1) IV; daf-12(rh61rh411) X* |  |  |  |  |
| FCZ428 | *nhr-80(tm1011) III; Punc-25-SNB::GFP (juIs1) IV* |  |  |  |  |
| FCZ444 | *nhr-8(hd117) Punc-25-SNB::GFP (juIs1) IV* |  |  |  |  |
| FCZ462 | *Punc-25-SNB::GFP (juIs1) IV; vit-2(ok3211) X* |  |  |  |  |
| FCZ463 | *Punc-25-SNB::GFP (juIs1) IV; vit-1(ok2616) X* |  |  |  |  |
| FCZ508 | *asm-3(ok1744) Punc-25-SNB::GFP (juIs1) IV* | *Punc-25-SNB::GFP* | Figure 2I |  |  |
| FCZ532 | *Punc-25-SNB::GFP (juIs1) IV; sms-2(tm2757) X* | *Punc-25-SNB::GFP* | Figure 2J |  | *sms-2(tm2757) is a gift from Guangshuo Ou.* |
| FCZ533 | *ncr-2(nr2023) III; Punc-25-SNB::GFP (juIs1) IV; sms-2(tm2757) X* |  |  |  |  |
| FCZ552 | *Punc-25-mcherry::Rab-3 (juIs236) II; sms-1(cfu52) IV* |  |  |  |  |
| FCZ553 | *Punc-25-mcherry::Rab-3 (juIs236) II; ncr-2(nr2023) III; sms-1(cfu52) IV* |  |  |  |  |
| FCZ554 | *sms-3(cfu62) III; Punc-25-SNB::GFP (juIs1) IV* |  |  |  |  |
| FCZ555 | *sms-3(cfu62) ncr-2(nr2023) III; Punc-25-SNB::GFP (juIs1) IV;* |  |  |  |  |
| FCZ544 | *sms-5(cfu66) II; Punc-25-SNB::GFP (juIs1) IV* |  |  |  |  |
| FCZ545 | *sms-5(cfu66) II; ncr-2(nr2023) III; Punc-25-SNB::GFP (juIs1) IV* |  |  |  |  |
| FCZ121 | *Punc-25::GFP(juIs76)II ; ncr-1(nr2022) X* | *Punc-25::GFP* | Figure 4A-4C |  |  |
| FCZ122 | *Punc-25::GFP(juIs76)II ; ncr-2(nr2023)Ⅲ* |  |  |  |  |
| FCZ123 | *Punc-25::GFP(juIs76)II ; ncr-2(nr2023) III ; ncr-1(nr2022) X* |  |  |  |  |
| FCZ233 | *Punc-25-SNB-1::GFP (juIs1) IV; ncr-1(nr2022) X; cfuEx28* | *cfuEx28/29/30: F25B3.3p::ncr-1 (genomic -116 to 8192 bp)* | Figure 5A-5C | pFC45 | Co-injection marker: *Pttx-3-RFP.*  Multiple lines were analyzed. |
| FCZ234 | *Punc-25-SNB-1::GFP (juIs1) IV; ncr-1(nr2022) X; cfuEx29* |  |  |  |  |
| FCZ235 | *Punc-25-SNB-1::GFP (juIs1) IV; ncr-1(nr2022) X; cfuEx30* |  |  |  |  |
| FCZ278 | *Punc-25-SNB-1::GFP (juIs1) IV; cfuEx53* | *cfuEx53/54/55:*  *F25B3.3p::ncr-1 (genomic -116 to 8192 bp)* |  |  |  |
| FCZ279 | *Punc-25-SNB-1::GFP (juIs1) IV; cfuEx54* |  |  |  |  |
| FCZ280 | *Punc-25-SNB-1::GFP (juIs1) IV; cfuEx55* |  |  |  |  |
| FCZ248 | *ncr-2(nr2023) III; Punc-25-SNB-1::GFP(juIs1) IV; ncr-1(nr2022) X; cfuEx38* | *cfuEx38/39/40:*  *F25B3.3p::ncr-1 (genomic -116 to 8192 bp)* | Figure S5B, S5D | pFC45 | Co-injection marker: *Pttx-3-RFP.*  Multiple lines were analyzed. |
| FCZ249 | *ncr-2(nr2023) III; Punc-25-SNB-1::GFP(juIs1) IV; ncr-1(nr2022) X; cfuEx39* |  |  |  |  |
| FCZ250 | *ncr-2(nr2023) III; Punc-25-SNB-1::GFP(juIs1) IV; ncr-1(nr2022) X; cfuEx40* |  |  |  |  |
| FCZ244 | *ncr-2(nr2023) III; Punc-25-SNB-1::GFP(juIs1) IV; ncr-1(nr2022) X; cfuEx37* | *cfuEx37:*  *vha-6p::ncr-1 (genomic -116 to 8192 bp)* | Figure S5B, S5D | pFC49 | Co-injection marker: *Pttx-3-RFP.* |
| FCZ236 | *Punc-25-SNB-1::GFP (juIs1) IV; ncr-1(nr2022) X; cfuEx31* | *cfuEx31/32/33/59/60/61/69:*  *sur-5p::ncr-1 (genomic -116 to 8192 bp)* | Figure 5A-5C | pFC53 | Co-injection marker: *Pttx-3-RFP.*  Multiple lines were analyzed. |
| FCZ237 | *Punc-25-SNB-1::GFP (juIs1) IV; ncr-1(nr2022) X; cfuEx32* |  |  |  |  |
| FCZ238 | *Punc-25-SNB-1::GFP (juIs1) IV; ncr-1(nr2022) X; cfuEx33* |  |  |  |  |
| FCZ284 | *Punc-25-SNB-1::GFP (juIs1) IV; cfuEx59* |  |  |  |  |
| FCZ285 | *Punc-25-SNB-1::GFP (juIs1) IV; cfuEx60* |  |  |  |  |
| FCZ286 | *Punc-25-SNB-1::GFP (juIs1) IV; cfuEx61* |  |  |  |  |
| FCZ306 | *Punc-25-SNB-1::GFP (juIs1) IV; cfuEx69* |  |  |  |  |
| FCZ239 | *ncr-2(nr2023) III; Punc-25-SNB-1::GFP(juIs1) IV; ncr-1(nr2022) X; cfuEx34* | *cfuEx34/35:*  *sur-5p::ncr-1 (genomic -116 to 8192 bp)* | Figure S5B, S5D | pFC53 | Co-injection marker: *Pttx-3-RFP.*  Multiple lines were analyzed. |
| FCZ240 | *ncr-2(nr2023) III; Punc-25-SNB-1::GFP(juIs1) IV; ncr-1(nr2022) X; cfuEx35* |  |  |  |  |
| FCZ251 | *Punc-25-SNB-1::GFP (juIs1) IV; ncr-1(nr2022) X; cfuEx41* | *cfuEx41/42/43/65/66:*  *myo-3p::ncr-1 (genomic -116 to 8192 bp)* | Figure 5A-5C | pFC51 | Co-injection marker: *Pttx-3-RFP.*  Multiple lines were analyzed. |
| FCZ252 | *Punc-25-SNB-1::GFP (juIs1) IV; ncr-1(nr2022) X; cfuEx42* |  |  |  |  |
| FCZ253 | *Punc-25-SNB-1::GFP (juIs1) IV; ncr-1(nr2022) X; cfuEx43* |  |  |  |  |
| FCZ298 | *Punc-25-SNB-1::GFP (juIs1) IV; cfuEx65* |  |  |  |  |
| FCZ299 | *Punc-25-SNB-1::GFP (juIs1) IV; cfuEx66* |  |  |  |  |
| FCZ254 | *ncr-2(nr2023) III; Punc-25-SNB-1::GFP(juIs1) IV; ncr-1(nr2022) X; cfuEx44* | *cfuEx44/45/46:*  *myo-3p::ncr-1 (genomic -116 to 8192 bp)* | Figure S5B, S5D | pFC51 | Co-injection marker: *Pttx-3-RFP.*  Multiple lines were analyzed. |
| FCZ255 | *ncr-2(nr2023) III; Punc-25-SNB-1::GFP(juIs1) IV; ncr-1(nr2022) X; cfuEx45* |  |  |  |  |
| FCZ256 | *ncr-2(nr2023) III; Punc-25-SNB-1::GFP(juIs1) IV; ncr-1(nr2022) X; cfuEx46* |  |  |  |  |
| FCZ369 | *ncr-2(nr2023) III; Punc-25-SNB-1::GFP(juIs1) IV; ncr-1(nr2022) X; cfuEx98* | *cfuEx98/99/100:*  *col-19p::ncr-1 (genomic -116 to 8192 bp)* | Figure S5B, S5D | pFC60 | Co-injection marker: *Pttx-3-RFP.*  Multiple lines were analyzed |
| FCZ370 | *ncr-2(nr2023) III; Punc-25-SNB-1::GFP(juIs1) IV; ncr-1(nr2022) X; cfuEx99* |  |  |  |  |
| FCZ371 | *ncr-2(nr2023) III; Punc-25-SNB-1::GFP(juIs1) IV; ncr-1(nr2022) X; cfuEx100* |  |  |  |  |
| FCZ378 | *ncr-2(nr2023) III; Punc-25-SNB-1::GFP(juIs1) IV; ncr-1(nr2022) X; cfuEx107* | *cfuEx107/108/109*  */110:*  *dpy-7p::ncr-1 (genomic -116 to 8192 bp)* | Figure S5B, S5D | pFC61 | Co-injection marker: *Pttx-3-RFP.*  Multiple lines were analyzed |
| FCZ379 | *ncr-2(nr2023) III; Punc-25-SNB-1::GFP(juIs1) IV; ncr-1(nr2022) X; cfuEx108* |  |  |  |  |
| FCZ380 | *ncr-2(nr2023) III; Punc-25-SNB-1::GFP(juIs1) IV; ncr-1(nr2022) X; cfuEx109* |  |  |  |  |
| FCZ381 | *ncr-2(nr2023) III; Punc-25-SNB-1::GFP(juIs1) IV; ncr-1(nr2022) X; cfuEx110* |  |  |  |  |
| FCZ308 | *ncr-2(nr2023) III; Punc-25-SNB-1::GFP(juIs1) IV; ncr-1(nr2022) X; cfuEx70* | *cfuEx70/71/72*  */73/74/75:*  *F25B3.3p::ncr-2 (genomic -16 to 7744 bp)* | Figure 5D-5F, S5C, S5D | pFC46 | Co-injection marker: *Pttx-3-RFP.*  Multiple lines were analyzed |
| FCZ309 | *ncr-2(nr2023) III; Punc-25-SNB-1::GFP(juIs1) IV; ncr-1(nr2022) X; cfuEx71* |  |  |  |  |
| FCZ310 | *ncr-2(nr2023) III; Punc-25-SNB-1::GFP(juIs1) IV; ncr-1(nr2022) X; cfuEx72* |  |  |  |  |
| FCZ311 | *Punc-25-SNB-1::GFP (juIs1) IV; cfuEx73* |  |  |  |  |
| FCZ312 | *Punc-25-SNB-1::GFP (juIs1) IV; cfuEx74* |  |  |  |  |
| FCZ313 | *Punc-25-SNB-1::GFP (juIs1) IV; cfuEx75* |  |  |  |  |
| FCZ317 | *ncr-2(nr2023) III; Punc-25-SNB-1::GFP(juIs1) IV; ncr-1(nr2022) X; cfuEx79* | *cfuEx76/77/78*  */79/80/81:*  *myo-3p::ncr-2 (genomic -16 to 7744 bp)* | Figure 5D-5F, S5C, S5D | pFC52 | Co-injection marker: *Pttx-3-RFP.*  Multiple lines were analyzed |
| FCZ318 | *ncr-2(nr2023) III; Punc-25-SNB-1::GFP(juIs1) IV; ncr-1(nr2022) X; cfuEx80* |  |  |  |  |
| FCZ319 | *ncr-2(nr2023) III; Punc-25-SNB-1::GFP(juIs1) IV; ncr-1(nr2022) X; cfuEx81* |  |  |  |  |
| FCZ314 | *Punc-25-SNB-1::GFP (juIs1) IV; cfuEx76* |  |  |  |  |
| FCZ315 | *Punc-25-SNB-1::GFP (juIs1) IV; cfuEx77* |  |  |  |  |
| FCZ316 | *Punc-25-SNB-1::GFP (juIs1) IV; cfuEx78* |  |  |  |  |
| FCZ324 | *ncr-2(nr2023) III; Punc-25-SNB-1::GFP(juIs1) IV; ncr-1(nr2022) X; cfuEx85* | *cfuEx85/86:*  *vha-6p::ncr-2 (genomic -16 to 7744 bp)* | Figure S5C, S5D | pFC50 | Co-injection marker: *Pttx-3-RFP.*  Multiple lines were analyzed |
| FCZ325 | *ncr-2(nr2023) III; Punc-25-SNB-1::GFP(juIs1) IV; ncr-1(nr2022) X; cfuEx86* |  |  |  |  |
| FCZ330 | *ncr-2(nr2023) III; Punc-25-SNB-1::GFP(juIs1) IV; ncr-1(nr2022) X; cfuEx90* | *cfuEx90/91:*  *col-10p::ncr-2 (genomic -16 to 7744 bp)* | Figure  S5C, S5D | pFC48 | Co-injection marker: *Pttx-3-RFP.*  Multiple lines were analyzed |
| FCZ331 | *ncr-2(nr2023) III; Punc-25-SNB-1::GFP(juIs1) IV; ncr-1(nr2022) X; cfuEx91* |  |  |  |  |
| FCZ273 | *ncr-2(nr2023) III; Punc-25-SNB-1::GFP(juIs1) IV; ncr-1(nr2022) X; cfuEx48* | *cfuEx48/49/50/51*  */52/62/63/64:*  *sur-5p::ncr-2 (genomic -16 to 7744 bp)* | Figure 5D-5F, S5C, S5D | pFC54 | Co-injection marker: *Pttx-3-RFP.*  Multiple lines were analyzed |
| FCZ274 | *ncr-2(nr2023) III; Punc-25-SNB-1::GFP(juIs1) IV; ncr-1(nr2022) X; cfuEx49* |  |  |  |  |
| FCZ275 | *ncr-2(nr2023) III; Punc-25-SNB-1::GFP(juIs1) IV; ncr-1(nr2022) X; cfuEx50* |  |  |  |  |
| FCZ276 | *ncr-2(nr2023) III; Punc-25-SNB-1::GFP(juIs1) IV; ncr-1(nr2022) X; cfuEx51* |  |  |  |  |
| FCZ277 | *ncr-2(nr2023) III; Punc-25-SNB-1::GFP(juIs1) IV; ncr-1(nr2022) X; cfuEx52* |  |  |  |  |
| FCZ295 | *Punc-25-SNB-1::GFP (juIs1) IV; cfuEx62* |  |  |  |  |
| FCZ296 | *Punc-25-SNB-1::GFP (juIs1) IV; cfuEx63* |  |  |  |  |
| FCZ297 | *Punc-25-SNB-1::GFP (juIs1) IV; cfuEx64* |  |  |  |  |
| FCZ420 | *ncr-2(nr2023) III; Punc-25-SNB-1::GFP(juIs1) IV; ncr-1(nr2022) X; cfuEx123* | *cfuEx123/124/125:*  *eak-4p::ncr-1 (genomic -116 to 8192 bp)* | Figure S5B, S5D | pFC69 | Co-injection marker: *Pttx-3-RFP.*  Multiple lines were analyzed |
| FCZ421 | *ncr-2(nr2023) III; Punc-25-SNB-1::GFP(juIs1) IV; ncr-1(nr2022) X; cfuEx124* |  |  |  |  |
| FCZ422 | *ncr-2(nr2023) III; Punc-25-SNB-1::GFP(juIs1) IV; ncr-1(nr2022) X; cfuEx125* |  |  |  |  |
| FCZ423 | *ncr-2(nr2023) III; Punc-25-SNB-1::GFP(juIs1) IV; ncr-1(nr2022) X; cfuEx126* | *cfuEx126/127/128:*  *eak-4p::ncr-2 (genomic -16 to 7744 bp)* | Figure S5C, S5D | pFC70 | Co-injection marker: *Pttx-3-RFP.*  Multiple lines were analyzed. |
| FCZ424 | *ncr-2(nr2023) III; Punc-25-SNB-1::GFP(juIs1) IV; ncr-1(nr2022) X; cfuEx127* |  |  |  |  |
| FCZ425 | *ncr-2(nr2023) III; Punc-25-SNB-1::GFP(juIs1) IV; ncr-1(nr2022) X; cfuEx128* |  |  |  |  |
| FCZ433 | *cfuEx132* | *cfuEx132/133/134: F25B3.3p::ncr-2::gfp+unc-119p::lmp-1::RFP* | Figure  6A, 6B | pFC66+  pFC76 | Co-injection marker: *Pttx-3-RFP.*  Multiple lines were analyzed. |
| FCZ434 | *cfuEx133* |  |  |  |  |
| FCZ435 | *cfuEx134* |  |  |  |  |
| FCZ436 | *cfuEx135* | *cfuEx135/136/137: F25B3.3p::ncr-1::gfp+unc-119p::lmp-1::RFP* | Figure  6A, 6B | pFC65+  pFC76 |  |
| FCZ437 | *cfuEx136* |  |  |  |  |
| FCZ438 | *cfuEx137* |  |  |  |  |
| FCZ509 | *cfuEx153* | *cfuEx153/154/155: dpy-7p::ncr-1::gfp+col-12p::lmp-1::RFP* | Figure  6A, 6C | pFC89+  pFC90 | Co-injection marker: *Pttx-3-RFP.*  Multiple lines were analyzed. |
| FCZ510 | *cfuEx154* |  |  |  |  |
| FCZ511 | *cfuEx155* |  |  |  |  |
| FCZ512 | *cfuEx156* | *cfuEx156/157/158: col-10p::ncr-2::gfp+col-12p::lmp-1::RFP* | Figure  6A, 6C | pFC89+  pFC84 |  |
| FCZ513 | *cfuEx157* |  |  |  |  |
| FCZ514 | *cfuEx158* |  |  |  |  |
| FCZ210 | *ncr-1(cfu39[ncr-1::gfp::loxp::3Xflag]) X* | CRISPR knock-in | Figure S6A, S6C, S6D, S6F | pFC36+ pFC37 |  |
| FCZ231 | *ncr-2(nr2023) Ⅲ; ncr-1::gfp (cfu39) X* |  |  |  |  |
| FCZ216 | *ncr-2(cfu45[ncr-2::gfp::loxp::3Xflag])* | CRISPR knock-in | Figure S6B, S6C, S6E and S6G | pFC41+ pFC42 |  |
| FCZ232 | *ncr-2::gfp(cfu45) III; ncr-1(nr2022) X* |  |  |  |  |
| FCZ637 | *ncr-1(nr2022) X; ldrIs2* | *ldrIs2[mdt-28p::mdt-28::mCherry + unc-76(+)]* | Figure 3A and 3B |  | *ldrIs2 is a gift from Pingsheng Liu and Bin Liang.* |
| FCZ638 | *ncr-2(nr2023) III; ldrIs2* |  |  |  |  |
| FCZ639 | *ncr-2(nr2023) III; ncr-1(nr2022) X; ldrIs2* |  |  |  |  |
| FCZ640 | *mboa-1(tm2464) X; ldrIs2* |  |  |  |  |
| FCZ641 | *daf-36(k114) V; ldrIs2* |  |  |  |  |
| FCZ654 | *ncr-2(nr2023) III; ncr-1(nr2022) mboa-1(tm2464)X; ldrIs2* |  |  |  |  |
| FCZ645 | *juIs1; krSi2* | *juIs1[Punc-25-SNB::GFP]*  *krSi2[Punc-49::unc49::tagRFP::unc-54 3'UTR]* | Figure  1F, 2E |  | *krSi2 is a gift from Haijun Tu.* |
| FCZ646 | *ncr-1(nr2022) X; juIs1; KrSi2* |  |  |  |  |
| FCZ647 | *ncr-2(nr2023) III; juIs1; KrSi2* |  |  |  |  |
| FCZ648 | *ncr-2(nr2023) III; ncr-1(nr2022) X; juIs1; KrSi2* |  |  |  |  |
| FCZ651 | *ncr-2(nr2023) III; ncr-1(nr2022) mboa-1(tm2464)X;* | *No marker* | Figure  3C-3G |  | *tm2464 is a gift from Shaobing Zhang.*  *e1370 is a gift from Xiaoxue Li.* |
| FCZ120 | *ncr-2(nr2023) III* |  |  |  |  |
| FCZ119 | *ncr-1(nr2022) X* |  |  |  |  |
| FCZ132 | *ncr-2(nr2023) III; ncr-1(nr2022) X* |  |  |  |  |
| SOZ1350 | *mboa-1(tm2464) X* |  |  |  |  |
| AA292 | *daf-36(k114) V* |  |  |  |  |
| CB1370 | *daf-2(e1370) III* |  |  |  |  |
