# Supplementary material for: Differential roles of lysosomal cholesterol transporters in the development of C. elegans NMJs
Source: Life Sci Alliance. 2024 Jul 31;7(10):e202402584. doi: 10.26508/lsa.202402584 (PMC11291935; doi:10.26508/lsa.202402584)
Supplement: Supplementary file 3 [file LSA-2024-02584_TableS2.docx]

**Table S2.** **Constructs for transgenes.**

| **Plasmid** | **Description** | **Notes** | **Allele #** |
| --- | --- | --- | --- |
| pFC45 | *F25B3.3p::ncr-1* | Genomic *ncr-1* sequence (including 116 bp upstream sequences before start codon and 847 bp downstream sequences after stop codon) | *cfuEx28/29/30*  *cfuEx38/39/40*  *cfuEx53/54/55* |
| pFC49 | *vha-6p::ncr-1* | Genomic *ncr-1* sequence (including 116 bp upstream sequences before start codon and 847 bp downstream sequences after stop codon) | *cfuEx36/37/47*  *cfuEx56/57/58* |
| pFC51 | *myo-3p::ncr-1* | Genomic *ncr-1* sequence (including 116 bp upstream sequences before start codon and 847 bp downstream sequences after stop codon) | *cfuEx41/42/43*  *cfuEx44/45/46*  *cfuEx65/66* |
| pFC53 | *sur-5p::ncr-1* | Genomic *ncr-1* sequence (including 116 bp upstream sequences before start codon and 847 bp downstream sequences after stop codon) | *cfuEx31/32/33*  *cfuEx34/35/59*  *cfuEx60/61/69* |
| pFC60 | *col-19p::ncr-1* | Genomic *ncr-1* sequence (including 116 bp upstream sequences before start codon and 847 bp downstream sequences after stop codon) | *cfuEx92/93/94*  *cfuEx95/96/97*  *cfuEx98/99/100* |
| pFC61 | *dpy-7p::ncr-1* | Genomic *ncr-1* sequence (including 116 bp upstream sequences before start codon and 847 bp downstream sequences after stop codon) | *cfuEx101/102/103*  *cfuEx104/105/106*  *cfuEx107/108/109*  *cfuEx110* |
| pFC69 | *eak-4p::ncr-1* | Genomic *ncr-1* sequence (including 116 bp upstream sequences before start codon and 847 bp downstream sequences after stop codon) | *cfuEx123/124/125* |
| pFC46 | *F25B3.3p::ncr-2* | Genomic *ncr-2* sequence (including 3 bp upstream sequences before start codon and 400 bp downstream sequences after stop codon) | *cfuEx70/71/72*  *cfuEx73/74/75* |
| pFC50 | *vha-6p::ncr-2* | Genomic *ncr-2* sequence (including 3 bp upstream sequences before start codon and 400 bp downstream sequences after stop codon) | *cfuEx82/83/84*  *cfuEx85/86* |
| pFC52 | *myo-3p::ncr-2* | Genomic *ncr-2* sequence (including 3 bp upstream sequences before start codon and 400 bp downstream sequences after stop codon) | *cfuEx76/77/78*  *cfuEx79/80/81* |
| pFC54 | *sur-5p::ncr-2* | Genomic *ncr-2* sequence (including 3 bp upstream sequences before start codon and 400 bp downstream sequences after stop codon) | *cfuEx48/49/50*  *cfuEx51/52*  *cfuEx62/63/64* |
| pFC48 | *col-10p::ncr-2* | Genomic *ncr-2* sequence (including 3 bp upstream sequences before start codon and 400 bp downstream sequences after stop codon) | *cfuEx87/88/89*  *cfuEx90/91* |
| pFC70 | *eak-4p::ncr-2* | Genomic *ncr-2* sequence (including 3 bp upstream sequences before start codon and 400 bp downstream sequences after stop codon) | *cfuEx126/127/128* |
| pFC65 | *F25B3.3p::ncr-1::GFP* | GFP is inserted after Leu1383. | *cfuEx138/139*  *cfuEx111/112/113*  *cfuEx135/136/137* |
| pFC90 | *dpy-7p::ncr-1::GFP* | GFP is inserted after Leu1383. | *cfuEx153/154/155* |
| pFC66 | *F25B3.3p::ncr-2::GFP* | GFP is inserted after Thr1269. | *cfuEx140/141/142*  *cfuEx129/130/131*  *cfuEx132/133/134* |
| pFC84 | *col-10p::ncr-2::GFP* | GFP is inserted after Thr1269. | *cfuEx156/157/158* |
| pFC76 | *unc-119p::lmp-1::RFP* | cDNA sequence of *lmp-1* (711 bp) | *cfuEx132/133/134*  *cfuEx135/136/137* |
| pFC89 | *col-12p::lmp-1::RFP* | cDNA sequence of *lmp-1* (711 bp) | *cfuEx153/154/155*  *cfuEx156/157/158* |
