# Supplementary material for: Differential roles of lysosomal cholesterol transporters in the development of C. elegans NMJs
Source: Life Sci Alliance. 2024 Jul 31;7(10):e202402584. doi: 10.26508/lsa.202402584 (PMC11291935; doi:10.26508/lsa.202402584)
Supplement: Supplementary file 4 [file LSA-2024-02584_TableS3.docx]

**Table S3. CRISPR knock-in & knock-out plasmids and genotyping primers**

| **Plasmid** | **Description** | **Notes** | **Gene (Allele)** |
| --- | --- | --- | --- |
| pFC36 | *Peft-3-cas9-NLS-pU6-sgRNA (ncr-1-658)* | sgRNA plasmid.  sgRNA sequence:  ATCTACAAAGATCGGAGATC | *ncr-1(cfu39[ncr-1::gfp::loxp::3Xflag])* |
| pFC37 | *pDD282-ncr-1::gfp* (2kb homology on each side of GFP) | *gfp* is inserted after Leu1383.  left recombination arm (2227 bp):  aagtaagatcagcgt…..gatctccgatctttg  right recombination arm(2474 bp):  tagataccgattt…..agaggtatggtccgg  genotyping primers:  FC257: ccttaaccgtccttctttactg  FC258: atctactagtctcttgtcatcgtc |  |
| pFC41 | *Peft-3-cas9-NLS-pU6-sgRNA (ncr-2-402)* | sgRNA plasmid.  sgRNA sequence: AGTGCTGGAAGTACTTGATG | *ncr-2(cfu45[ncr-2::gfp::loxp::3Xflag])* |
| pFC42 | *pDD282-ncr-2::gfp*  (2kb homology on each side of GFP) | *gfp* is inserted after Thr1269.  left recombination arm (2128 bp):  attgaagatgatag…..aagtacttccagc  right recombination arm (2049 bp):  acttctgaaacat…. gtgctaaatggatc  genotyping primers:  FC271: catttgctgattctcgtgtg  FC272: cgggctaagactattctgag |  |
| pFC86 | *Peft-3-cas9-NLS-pU6-sgRNA (sms-1#1)* | sgRNA plasmid.  sgRNA sequence:  GCCCCGTTGCTACCTGACGA | *sms-1(cfu52)* deletes 31bp (TGCTACCT GACGAAGGACCATCGCATGCCGT).  PCR primers:  FC462:  gccaagacaatttgttcgatgga  FC463: gaacacaatatcaggaagtggtgg  Sequencing primers:  FC463 |
| pFC87 | *Peft-3-cas9-NLS-pU6-sgRNA (sms-1#2)* | sgRNA plasmid.  sgRNA sequence:  ACATTCTTCACTGGCTGTCA |  |
| pFC97 | *Peft-3-cas9-NLS-pU6-sgRNA (sms-3#1)* | sgRNA plasmid.  sgRNA sequence:  TTAGCCTGGTTTCGGAGCAA | *sms-3(cfu62)* deletes 1324bp (TTAGCCTGGTTTCGGAGCAA...ATGCCGGGATCAAGTGGAGT).  genotyping primers:  FC517:  gtgagcaagacagtaataagtg FC518: aaattgagctagaacctcctg  FC601:  aactcacctaaactagaagcc |
| pFC98 | *Peft-3-cas9-NLS-pU6-sgRNA (sms-3#2)* | sgRNA plasmid.  sgRNA sequence:  GCGCTATGCATCGTGATGCT |  |
| pFC99 | *Peft-3-cas9-NLS-pU6-sgRNA (sms-3#3)* | sgRNA plasmid.  sgRNA sequence:  CTCGTCATCCATCAGCACCG |  |
| pFC100 | *Peft-3-cas9-NLS-pU6-sgRNA (sms-3#4)* | sgRNA plasmid.  sgRNA sequence:  CGGGGTACACGGATAATCAG |  |
| pFC93 | *Peft-3-cas9-NLS-pU6-sgRNA (sms-5#1)* | sgRNA plasmid.  sgRNA sequence:  ACTCTAGATACTCAATAGAC | *sms-5(cfu66)* deletes CAAAT in the sequences (aaattttctagGGCGTCGGTTGGCTATCAAATGAGGTTGCATTGGCTTGG).  PCR primers:  FC541:  ctacgaacacgatgagcagag FC542: cggagtcattgaaattggctgag  Sequencing primers:  FC547:  tcaaaaaactagctggggacc |
| pFC94 | *Peft-3-cas9-NLS-pU6-sgRNA (sms-5#2)* | sgRNA plasmid.  sgRNA sequence:  CGTCGGTTGGCTATCAAATG |  |
| pFC95 | *Peft-3-cas9-NLS-pU6-sgRNA (sms-5#3)* | sgRNA plasmid.  sgRNA sequence:  ATCAGCACCGATGGATAGTT |  |
| pFC96 | *Peft-3-cas9-NLS-pU6-sgRNA (sms-5#4)* | sgRNA plasmid.  sgRNA sequence:  GTGAAAATGTTCTGGAGCGC |  |
